# Imatinib relaxes the pulmonary venous bed of guinea pigs

**DOI:** 10.1186/s12931-017-0514-0

**Published:** 2017-02-08

**Authors:** Nina A. Maihöfer, Said Suleiman, Daniela Dreymüller, Paul W. Manley, Rolf Rossaint, Stefan Uhlig, Christian Martin, Annette D. Rieg

**Affiliations:** 1 Institute of Pharmacology and Toxicology, Medical Faculty Aachen, RWTH-Aachen, Aachen, Germany; 2 Novartis Pharma, AG Basel, Switzerland; 3 Department of Anesthesiology, Medical Faculty Aachen, RWTH-Aachen, Aachen, Germany

**Keywords:** Pulmonary veins, Postcapillary resistance, Pulmonary hypertension due to left heart disease, Imatinib, Tyrosine kinase inhibitors, PDGFR

## Abstract

**Background:**

Recently, the IMPRES study revealed that systemic imatinib improves exercise capacity in patients with advanced pulmonary arterial hypertension. Imatinib blocks the tyrosine kinase activity of the platelet-derived growth factor (PDGF)-receptor (PDGFR), acts antiproliferative and relaxes pulmonary arteries. However so far, the relaxant effects of imatinib on pulmonary veins (PVs) and on the postcapillary resistance are unknown, although pulmonary hypertension (PH) due to left heart disease (LHD) is most common and primarily affects PVs. Next, it is unknown whether activation of PDGFR alters the pulmonary venous tone. Due to the reported adverse effects of systemic imatinib, we evaluated the effects of nebulized imatinib on the postcapillary resistance.

**Methods:**

Precision-cut lung slices (PCLS) were prepared from guinea pigs. PVs were pre-constricted with Endothelin-1 (ET-1) and the imatinib-induced relaxation was studied by videomicroscopy; PDGF-BB-related vascular properties were evaluated as well. The effects of perfused/nebulized imatinib on the postcapillary resistance were studied in cavine isolated perfused lungs (IPL). Intracellular cAMP/cGMP was measured by ELISA in PVs.

**Results:**

In PCLS, imatinib (100 μM) relaxed pre-constricted PVs (126%). In PVs, imatinib increased cAMP, but not cGMP and inhibition of adenyl cyclase or protein kinase A reduced the imatinib-induced relaxation. Further, inhibition of K_ATP_-channels, ${\mathrm{BK}}_{\mathrm{Ca}}^{2+}$-channels or K_v_-channels diminished the imatinib-induced relaxation, whereas inhibition of NO-signaling was without effect. In the IPL, perfusion or nebulization of imatinib reduced the ET-1-induced increase of the postcapillary resistance. In PCLS, PDGF-BB contracted PVs, which was blocked by imatinib and by the PDGFR-β kinase inhibitor SU6668, whereas inhibition of PDGFR-α (ponatinib) had no significant effect. Conversely, PDGFR-β kinase inhibitors (SU6668/DMPQ) relaxed PVs pre-constricted with ET-1 comparable to imatinib, whereas the PDGFR-α kinase inhibitor ponatinib did not.

**Conclusions:**

Imatinib-induced relaxation depends on cAMP and on the activation of K^+^-channels. Perfused or nebulized imatinib significantly reduces the postcapillary resistance in the pre-constricted (ET-1) pulmonary venous bed. Hence, nebulization of imatinib is feasible and might reduce systemic side effects. Conversely, PDGF-BB contracts PVs by activation of PDGFR-β suggesting that imatinib-induced relaxation depends on PDGFR-β-antagonism. Imatinib combines short-term relaxant and long-term antiproliferative effects. Thus, imatinib might be a promising therapy for PH due to LHD.

## Background

Pulmonary hypertension (PH) due to left heart disease (LHD) is the most common cause of PH [1–5]. In response to elevated left ventricular (LV) or atrial filling pressure, PH due to LHD is associated with increased pulmonary capillary wedge pressure (P_pcw_) >15 mmHg leading by backwards transmission to elevated pulmonary arterial pressure (P_PA_) ≥25 mmHg, even though the transpulmonary gradient (TPG) often remains ≤12 mmHg [6]. Though, some patients develop severe PH characterized by a TPG ≥12, by proliferative pulmonary vascular disease and elevated pulmonary vascular resistance (PVR) [6]. PH due to LHD primarily affects the pulmonary veins (PVs) [7], thus it is also called postcapillary PH or pulmonary venous hypertension (PVH) [1, 4, 8]. This feature is pivotal for the therapy of PH due to LHD, as vasodilators acting primarily in the pulmonary arterial bed may increase pulmonary perfusion leading to elevated hydrostatic pressure, pulmonary edema and elevated PVR [8]. Until now, specific agents to treat PH due to LHD are missing, although some agents, e.g. the PDE-III inhibitor milrinone exert relaxant effects in human PVs [9]. Thus, current guidelines recommend the management of the underlying condition, e.g. the therapy of LHD, the repair of valvular heart disease or LV-assist device implantation [3, 6]. However, cardiac surgery is at high risk. Therefore, new therapeutic approaches are warranted.

The tyrosine kinase inhibitor (TKI) imatinib represents such a novel approach which demonstrated already promise in experimental models [10] and clinical trials [11–13]. Moreover, two studies in rats show that imatinib may act also as a vasodilator in rat pulmonary arteries (PAs) and aortas [14, 15]. Recently, the IMPRES study showed that oral imatinib improves exercise capacity and hemodynamics in patients with advanced pulmonary arterial hypertension (PAH) [16]. So far, although relevant, the effects of imatinib have been never studied in the pulmonary venous bed or in PH due to LHD. Of note, the IMPRES study uncovered also severe imatinib-related adverse effects such as subdural hematoma or QTc prolongation [16, 17]. Thus, a local application, e.g. by nebulization could be advantageous to improve the safety of imatinib [18].

Imatinib resembles an intriguing approach in PH, as it targets the vascular remodeling, a major pathophysiologic condition of PH. It inhibits the receptor tyrosine kinase (RTK) platelet derived growth factor (PDGF)-receptor (PDGFR) and counteracts thereby PDGF [19, 20]. Both PDGF and its receptor are overexpressed in pulmonary arterial smooth muscle cells (SMCs) and to lesser extent in endothelial cells of patients with idiopathic PAH [21]. Further, PDGFR-β immunoreactivity is increased in small arterioles and venules, as well as in capillaries of patients with pulmonary venous occlusive disease and systemic sclerosis associated PAH [22]. PDGFR consists of two subunits (αα, αβ, ββ) among which PDGFR-β mediates proliferation [23]. At present it is not known whether PDGF alters the pulmonary vascular tone. In systemic vessels, PDGF acts as a vasodilator in mesenteric arteries [24, 25] and as a vasoconstrictor in aortas [26]. However, these discrepant findings do not allow to predict the action of PDGF in the lungs, in particular, because the pulmonary and the systemic vasculature are remarkably dissimilar [27].

With regard to PH due to LHD, there are several unsolved issues concerning the effects of imatinib and PDGF on the pulmonary venous tone. 1) Does imatinib relax PVs? 2) Which are the possible underlying mechanisms? 3) Does imatinib affect the postcapillary resistance (R_post_)? 4) Does imatinib exert pulmonary relaxant effects when applied by nebulization? 5) Does PDGF act as a pulmonary vasoconstrictor? — A finding that would in part explain the relaxant effects of imatinib. In the present study, we address these questions to evaluate the value of imatinib within the context of PH due LHD.

## Methods

### Animals

Female Dunkin Hartley GPs (350 ± 50 g) were purchased from Charles River (Sulzfeld, Germany). Animal studies were approved by the Landesamt für Natur, Umwelt und Verbraucherschutz Nordrhein-Westfalen (ID: 8.87-51.05.20.10.245) and performed following the Directive 2010/63/EU of the European Parliament.

### PCLS

PCLS were prepared as described [28, 29]. Briefly, intraperitoneal anesthesia was performed with 95 mg kg^−1^ pentobarbital (Narcoren; Garbsen, Germany) and verified by missing reflexes. The GP was bled, the trachea cannulated and the diaphragm opened. The lungs were filled via the trachea with 1.5% low melting agarose. To harden the lungs, they were cooled with ice. Then, tissue cores (diameter 11 mm) were prepared and cut into about 300 μM thick slices with a Krumdieck tissue slicer (Alabama Research & Development, Munford, AL, USA). PCLS were incubated at 37 °C and repeated medium changes were performed to wash out the agarose.

### Cyclic AMP and cGMP enzyme immunoassay

To analyze cAMP/cGMP, PVs were isolated out of tissue cores guided by their anatomical landmarks, e.g. the PAs accompany the airways, whereas PV lies aside. PVs were incubated in medium, flushed with imatinib (100 μM) and after 30 min frozen by liquid nitrogen. Cyclic AMP/cGMP was quantified with ELISA-kits following the manufacturer’s protocol. For stabilization, all samples or standards were acetylated. To measure cAMP, all samples were diluted 1:2 with 0.1 M HCL. The ELISA was evaluated at 405 nM (GENIOS, Tecan, Switzerland).

### Measurements, pharmacological interventions and videomicroscopy

**Fig. 1:**
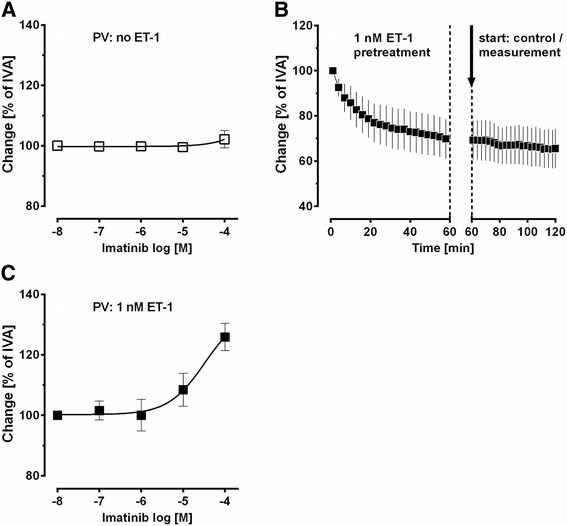
Vascular effects of imatinib and ET-1 in pulmonary veins of GPs. **a** Concentration-response curve of imatinib in naïve PVs: (□) PV (*n* = 5). **b** Treatment of naïve PVs with 1 nM ET-1 to obtain a stable pre-constriction: (■) PV 1 nM ET-1 (*n* = 6). The dashed line indicates the end of pre-treatment. **c** Effect of imatinib in ET-1-pre-constricted PVs: (■) PV (*n* = 9)

To study the relaxant effects of imatinib, PVs were incubated with 1 nM ET-1 (Fig. 1b) to induce a stable contraction after 1 h. If a signaling pathway was evaluated, PCLS were additionally pre-treated for 1 h with one of the following inhibitors at concentrations about 10–100 fold above the IC_50_ value of the target: Adenyl cyclase: 100 μM SQ22536 (IC_50_: 1.4 – 200 μM) [30]; protein kinase A (PKA): 1 μM KT5720 (IC_50_: 60 nM); NO-synthase: 100 μM L-NAME (IC_50_: 25 μM); protein kinase G (PKG): 2 μM KT5823 (IC_50_: 0.23 μM); K_ATP_-channel: 10 μM glibenclamide (IC_50_: 20 – 200 nM); BK_Ca_^2+^-channel: 100 nM iberiotoxin (IC_50_: 10 nM); K_v_-channel: 5 mM 4-aminopyridine (IC_50_: 0.3 – 1.1 mM) [31]; PDGFR-α: 100 nM ponatinib (IC_50_: 1.1 nM) [32–34]; PDGFR-β: 5 μM SU6668 (IC_50_: 0.008 – 0.1 μM) [35–37] or 5 μM DMPQ (IC_50_: 0.08 μM). Subsequently, concentration-response curves with imatinib, ponatinib, SU6668 or DMPQ were performed. To study the contractile effect of PDGF-BB, PVs were pretreated with 100 μM imatinib (PDGFR); 5 μM SU6668 (PDGFR-β) or 100 nM ponatinib (PDGFR-α). In PCLS, all changes of the initial vessel area (IVA) were quantified in % and are reported as “Change [% of IVA]”. Hence, a vessel area <100% indicates contraction and a vessel area >100% indicates relaxation. To compare relaxation of pre-treated vessels, the vessel area was defined after pre-treatment again as 100%. Concentration-response curves of the vasodilators were performed and the effects reported as “Change [% of IVA]”. In the graphs, all pre-treatments were indicated. The luminal area of PVs was monitored with a digital video camera (Leica Viscam 1280, Leica DFC 280). The images were analyzed with Optimas 6.5 (Media Cybernetics, Bothell, WA).

### Isolated perfused lungs of the GP

Cavine lungs were prepared as described [9, 38]. Briefly, intraperitoneal anesthesia was performed (pentobarbital: 95 mg kg^−1^) and verified by missing reflexes. The GP was bled, the trachea cannulated and the lung ventilated with positive pressure (70 breaths/min). The left ventricle’s apex was cut and cannulas were placed in the pulmonary artery (perfusion inflow) and in the left atrium (perfusion outflow). The lung was perfused at constant flow (12,5 mL/min) with Krebs-Henseleit buffer, containing 2% bovine serum albumin, 0.1% glucose, 0.3% HEPES and 50 nM salbutamol to prevent bronchoconstriction [39]. The temperature of the perfusate was maintained at 37 °C with a water bath and the pH was maintained between 7.35 and 7.45 by gassing with carbon dioxide. Heart and lungs were removed and transferred into a negative-pressure chamber. Tidal volume, compliance, resistance, pulmonal arterial pressure (P_PA_), left atrial pressure (P_LA_), and the flow were continuously monitored. As soon as the respiratory and hemodynamic variables remained stable over 10 min, ET-1 (20 nM) was added to the recirculating perfusion buffer (total volume 200 mL) to enhance R_post_ [40]. Ten minutes after the application of ET-1, imatinib (10 μM) was perfused. At a buffer volume of 200 mL, this corresponds to total amount of 1.18 mg imatinib or to 3.5 mg/kg body weight imatinib, respectively. Thereafter, changes of the capillary pressure (P_cap_) were measured every 10 min by the double occlusion method [38], R_post_ and the precapillary resistance (R_pre_) were calculated by the following equations: ${\mathrm{R}}_{\mathrm{post}}=\frac{Pcap- PLA}{Flow}$ and ${\mathrm{R}}_{\mathrm{pre}}=\frac{PPA- Pcap}{Flow}$.

In order to examine the effects of nebulized imatinib on R_post_, isolated perfused GP lungs were perfused as described before. After the increase of R_post_ by ET-1 (20 nM), 3 mL of imatinib (10 mM), corresponding to a total amount of 17,691 mg imatinib were nebulized over a period of 130 min. Assuming a lung flow of 0,21 L/min (70 breaths à 3 mL) and a pressure of 1.5 bar, the total amount of inhaled imatinib corresponds to less than 4% of the nebulized amount of imatinib [41], namely 0,71 mg, corresponding to 2 mg/kg body weight imatinib, respectively. The contractile effect of ET-1 agonists strongly varies. Therefore, the maximal ET-1-induced increase of R_post_ was normalized to 100% and all submaximal increases of R_post_ were indicated in % of the maximal increase of R_post_.

### Chemicals

Imatinib was provided by Novartis (Basel, Switzerland); nebulized imatinib was solved in aqua at a concentration of 10 mM. SQ22536, KT5720, KT5823, glibenclamide, iberiotoxin, 4-aminopyridine and DMPQ were purchased from Tocris Bioscience (Ellisville, Missouri, USA). ET-1 was acquired from BIOTRENDS (Wangen, Switzerland) and SU6668 and ponatinib were acquired from Biomol (Hamburg, Germany). L-Name or standard laboratory chemicals were obtained from Sigma-Aldrich (Steinheim, Germany). The ELISA-kits were acquired from Enzo (Lörrach, Germany). Human PDGF-BB was delivered by Peprotech (Hamburg, Germany).

### Statistical analysis

**Fig. 2:**
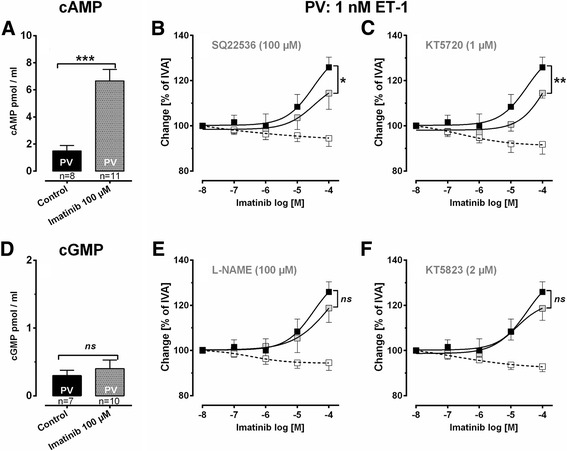
Role of cAMP/cGMP in imatinib-induced relaxation of GP pulmonary veins. **a** Effect of imatinib on intracellular cAMP. **b** Effect of inhibition of adenyl cyclase (SQ22536) on imatinib-induced relaxation in ET-1 (1 nM) pre-constricted PVs: (■) imatinib (*n* = 9); () SQ22536 (100 μM), imatinib (*n* = 7); (□) SQ22526 (100 μM) (*n* = 6). **c** Effect of inhibition of PKA (KT5720) on imatinib-induced relaxation in ET-1 (1 nM) pre-constricted PVs: (■) imatinib (*n* = 9); () KT5720 (1 μM), imatinib (*n* = 7); (□) KT5720 (1 μM) (*n* = 6). **d** Effect of imatinib on cGMP. **e** Effect of inhibition of NO-synthase (L-NAME) on imatinib-induced relaxation in ET-1 (1 nM) pre-constricted PVs: (■) imatinib (*n* = 9); () L-NAME (100 μM), imatinib (*n* = 8); (□) L-NAME (100 μM) (*n* = 5). **f** Effect of inhibition of PKG (KT5823) on imatinib-induced relaxation in ET-1 (1 nM) pre-constricted PVs: (■) imatinib (*n* = 9); () KT5823 (2 μM), imatinib (*n* = 11); (□) KT5823 (2 μM) (*n* = 6). A/D) Statistics was performed by the Mann–Whitney *U* test. **b**/**c**/**e**/**f**) Asterisks indicate different EC_50_ values. *P* <0.05 are considered as significant: * *p* <0.05, ** *p* <0.01 and *** *p* <0.001

**Fig. 3:**
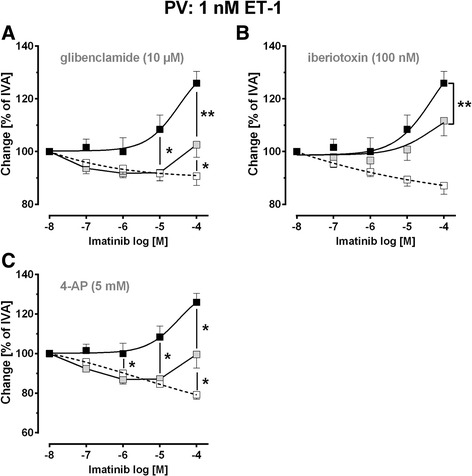
Contribution of K^+^-channels to imatinib-induced relaxation of GP pulmonary veins. **a** Effect of inhibition of K_ATP_-channels (glibenclamide) on imatinib-induced relaxation in ET-1 (1 nM) pre-constricted PVs: (■) imatinib (*n* = 9); () glibenclamide (10 μM), imatinib (*n* = 8); (□) glibenclamide (10 μM) (*n* = 8); **b** Effect of inhibition of ${\mathrm{BK}}_{\mathrm{Ca}}^{2+}$-channels (iberiotoxin) on imatinib-induced relaxation in ET-1 (1 nM) pre-constricted PVs: (■) imatinib (*n* = 9); () iberiotoxin (100 nM), imatinib (*n* = 8); (□) iberiotoxin (100 nM) (n = 8); **c** Effect of inhibition of K_v_-channels (5-aminopyridine) on imatinib-induced relaxation in ET-1 (1 nM) pre-constricted PVs: (■) imatinib (*n* = 9); () 4-AP (5 mM), imatinib (*n* = 10); (□) 4-AP (5 mM) (*n* = 10). **a**/**c** Corresponding concentrations of (■) and () were compared by the Mann–Whitney *U* test. **b** Asterisks indicate different EC_50_ values. *P* <0.05 are considered as significant: * *p* <0.05 and ** *p* <0.01

**Fig. 4:**
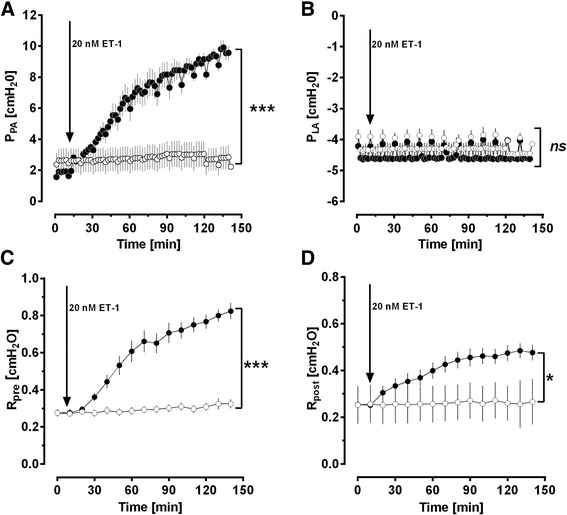
Influence of ET-1 on different segments of the pulmonary circulation in the IPL. **a** Influence of 20 nM ET-1 on the pulmonary arterial pressure (P_PA_): (○) control (*n* = 6); (●) ET-1 20 nM (*n* = 7); **b** Influence of 20 nM ET-1 on the left atrial pressure (P_LA_): (○) control (*n* = 6); (●) ET-1 20 nM (*n* = 7); **c** Influence of 20 nM ET-1 on the precapillary resistance (R_pre_): (○) control (*n* = 6); (●) ET-1 20 nM (*n* = 7); **d** Influence of 20 nM ET-1 on the postcapillary resistance (R_post_): (○) control (*n* = 6); (●) ET-1 20 nM (*n* = 7). **a**-**d**) Statistics was performed by a LMM. *P* <0.05 are considered as significant: * *p* <0.05, ** *p* <0.01 and *** *p* <0.001

**Fig. 5:**
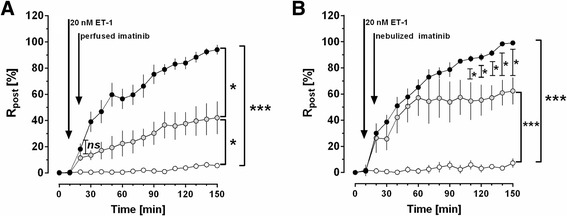
Influence of perfused and nebulized imatinib on the ET-1-induced increase of R_post_. **a** Influence of perfused imatinib on the ET-1-induced increase of the postcapillary resistance (R_post_): (○) control (*n* = 6); (●) ET-1 20 nM (*n* = 7); () ET-1 20 nM / imatinib 20 mM (*n* = 6). **b** Influence of nebulized imatinib on the ET-1-induced increase of the postcapillary resistance (R_post_): (○) control (*n* = 6); (●) ET-1 20 nM (*n* = 7); () ET-1 20 nM/imatinib 20 mM (*n* = 7). **a**-**b** Statistics was performed by a LMM. *P* <0.05 are considered as significant: * *p* <0.05 and *** *p* <0.001

**Fig. 6:**
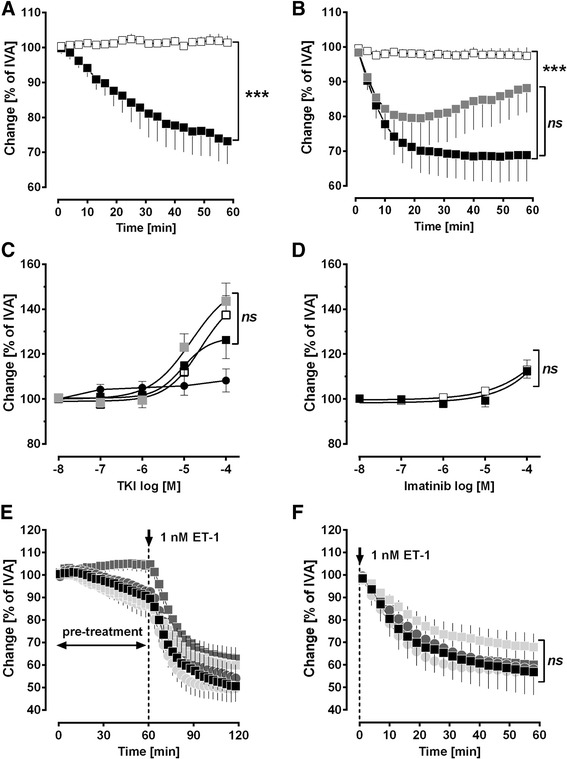
The role of PDGFR-β and interaction of ET-1 with PDGFR. **a** Effect of inhibition of PDGFR (imatinib) on the contractile effect of 10 nM PDGF-BB: (■) PV: PDGF-BB (10 nM) (*n* = 5); (□) PV: imatinib (100 μM), PDGF-BB (*n* = 5). **b** Effect of inhibition of PDGFR-α (ponatinib) and PDGFR-β (SU6668) on the contractile effect of PDGF-BB: (■) PV: PDGF-BB (100 nM) (*n* = 7); (□) PV: SU6668 (5 μM), PDGF-BB (100 nM) (*n* = 6); () PV: Ponatinib (100 nM), PDGF-BB (100 nM) (*n* = 7). **c** The relaxant effects of the unselective TKI imatinib and the PDGFR-β inhibitors SU6668 or DMPQ in ET-1 pre-constricted PVs: (■) PV 1 nM ET-1/imatinib (*n* = 5); () PV: 1 nM ET-1/SU6668 (*n* = 5); (□) PV: 1 nM ET-1/DMPQ (*n* = 5); (●) PV: 1 nM ET-1/ponatinib (*n* = 5); **d** The relaxant effect of the unselective TKI imatinib after inhibition of PDGFR-β by SU6668 or DMPQ: (■) PV: 5 μM SU6668/1 nM ET-1/imatinib; (□) PV: 5 μM DMPQ/1 nM ET-1/imatinib; **e**/**f** Effect of inhibition of PDGFR (imatinib), PDGFR-α (ponatinib) and PDGFR-β (SU6668) on ET-1 induced contraction: (■) PV: ET-1 (1 nM) (*n* = 5); () PV: 100 μM imatinib/1 nM ET-1 (*n* = 5); () PV: 1 μM imatinib/1 nM ET-1 (*n* = 5); () PV: 5 μM SU6668/1 nM ET-1 (*n* = 5); () PV: 100 nM ponatinib/1 nM ET-1 (*n* = 5). Statistics was performed by LMM (Fig. 6 **a**, **b**, **e**, **f**). Asterics indicate different EC_50_ values (Fig. 6 **c**, **d**). *P* <0.05 are considered as significant: *** < 0.001

Statistics were conducted using SAS software 9.3 (SAS Institute, Cary, North Carolina, USA) and GraphPad Prism 5.01 (GraphPad, La Jolla, USA). The data in Figs. 4, 5 and 6a,b,e,f were analyzed using a linear mixed model analysis (LMM) with the covariance structures VC or AR(1); EC_50_ values (Figs. 2b, c, e, f, 3b and 6c, d) were calculated by the standard 4-parameter logistic non-linear regression model (GraphPad, La Jolla, USA). The AIC-criterion was used to select the most parsimonious model, i.e. a common top, bottom, slope and EC_50_ value in the regression model or the covariance matrix with the least number of parameters. Non-parametric analysis (Figs. 2a, d and 3a, c) was performed by the Mann–Whitney *U* test. All p-values were adjusted for multiple comparisons by the false discovery rate and are presented as mean ± SEM; n indicates the numbers of animals. *P* <0.05 was considered as significant.

## Results

We studied the relaxant effects of imatinib in naïve (not pre-constricted) and in pre-constricted PVs.

### ET-1-induced pre-constriction and imatinib-induced relaxation

Imatinib did not relax naïve PVs from GPs (Fig. 1a). To obtain a stable and comparable contraction PVs were pre-constricted with ET-1 (1 nM). After 1 h, ET-1 (1 nM) contracted PVs to 69% of IVA (Fig. 1b), and imatinib (100 μM) relaxed PVs to 126% of IVA (Fig. 1c).

### Involvement of the cAMP/PKA-pathway to the vasorelaxant effect of imatinib

In PVs, imatinib increased intracellular cAMP (Fig. 2a). The functional role of the cAMP/PKA-pathway was addressed by pre-treatments with the adenyl cyclase-inhibitor SQ22536 (100 μM) and the PKA-inhibitor KT5720 (1 μM). Both inhibitors alone do not alter the contractile effect of ET-1 [29], but here they decreased the imatinib-induced relaxation (Fig. 2b, c). To study the contribution of NO or NO-downstream products (cGMP/PKG) to the imatinib-induced relaxation, PVs were pre-treated with the NO-synthase-inhibitor L-NAME (100 μM) or the PKG-inhibitor KT5823 (2 μM). Both inhibitors do not affect the ET-1-induced contraction [29]. Here, L-NAME and KT5823 did also not alter the relaxant effect of imatinib (Fig. 2e, f). Accordingly, imatinib did not increase intracellular cGMP-levels in PVs (Fig. 2d).

### Involvement of K^+^-channels to the vasorelaxant effect of imatinib

We further evaluated the impact of K^+^-channel-activation in the imatinib-induced relaxation of cavine PVs. ${\mathrm{K}}_{\mathrm{ATP}}$-channel-inhibition by glibenclamide (10 μM), ${\mathrm{BK}}_{\mathrm{Ca}}^{2+}$-channel-inhibition by iberiotoxin (100 nM) and ${\mathrm{K}}_{\mathrm{v}}$-channel-inhibition by 5 mM 4-aminopyridine (4-AP) do not affect ET-1-induced contraction [29]. However, inhibition of ${\mathrm{K}}_{\mathrm{ATP}}$-channels, ${\mathrm{BK}}_{\mathrm{Ca}}^{2+}$-channels or ${\mathrm{K}}_{\mathrm{v}}$-channels significantly reduced the relaxant effect of imatinib (Fig. 3a-c); inhibition of ${\mathrm{K}}_{\mathrm{ATP}}$- or ${\mathrm{K}}_{\mathrm{v}}$-channels was most effective.

### Imatinib lowers the R_post_ in the IPL

To elucidate whether and how imatinib affects the pulmonary venous bed in an intact organ, we perfused and nebulized imatinib in the IPL after raising R_post_ by ET-1. In the IPL, perfusion of 20 nM ET-1 increased P_PA_ about 600% of baseline from 1.5 cmH_2_O to 9.5 cmH_2_O (*p* <0.0001), whereas control lungs had no increase of P_PA_ and remained stable (Fig. 4a). ET-1 did not affect P_LA_ (Fig. 4b). Further, the experiments with the double occlusion method revealed that ET-1 significantly enhanced R_pre_ up to 312% and R_post_ up to 169% of baseline (Fig. 4c, d). Next we studied if perfused or nebulized imatinib significantly affects R_post_. Perfusion of imatinib significantly lowered the ET-1-induced increase of R_post_ (Fig. 5a), even though R_post_ was still increased (*p* <0.05) compared to control lungs (Fig. 5a). Nebulization of imatinib (calculated dosage: 2 mg/kg body weight) reduced the ET-1-induced increase of R_post_ from the time points 110 – 150 min, but R_post_ was still significantly increased (*p* <0.001) compared to control lungs (Fig. 5b).

### The role of PDGFR-β and interaction of ET-1 with PDGFR

The antiproliferative properties of imatinib are likely explained by PDGFR-inhibition. Thus, we hypothesized that the imatinib-induced relaxation may depend on inhibition of PDGFR kinase activity and further that PDGF-BB may possibly exert contractile properties in pulmonary vessels. To test this hypothesis and to identify, which PDGFR-subunit might be responsible for a possible contractile effect, we pre-treated PVs with 100 μM imatinib (PDGFR), 5 μM SU6668 (PDGFR-β) or 100 nM ponatinib (PDGFR-α) prior to the treatment with 10 or 100 nM PDGF-BB. Control PVs treated with 10 or 100 nM PDGF-BB contracted up to 70% of IVA. In contrast, pre-treatment with imatinib and SU6668 abolished the contractile effect of PDGF-BB, whereas pre-treatment with ponatinib only insignificantly reduced the contractile effect of PDGF-BB (Fig. 6a, b). Next, we analyzed the effects of the PDGFR-α inhibitor ponatinib and the PDGFR-β inhibitors SU6668 and DMPQ on PVs pre-constricted with ET-1. Treatment with SU6668 and DMPQ relaxed PVs pre-constricted with ET-1 up to 140% of IVA (Fig. 6c), whereas treatment with the PDGFR-α inhibitor ponatinib had no significant relaxant effect (Fig. 6c). Further, concomitant pre-treatment with 5 μM SU6668 and 1 nM ET-1 or 5 μM DMPQ and 1 nM ET-1 (Fig. 6d) abolished imatinib-induced relaxation, exceptional if it was applied at 100 μM. Next, we studied whether stimulation of ET-1 receptors interacts anyhow with PDGFR or conversely, if activation of PDGFR contributes to the contractile effect of ET-1. In order to inhibit PDGFR, we pre-treated PVs 60 min with imatinib (10 or 100 μM), 5 μM SU6668 or 100 nM ponatinib prior to the treatment of 1 nM ET-1 (Fig. 6e, f). We found, irrespective of inhibition of PDGFR-αβ, ET-1 contracted PVs without significant difference (Fig. 6e, f).

## Discussion

The tyrosine kinase inhibitor imatinib represents an intriguing approach to treat PH. Initially, research focused on the antiproliferative effects of imatinib, in line with the reversal of vascular remodeling in experimental studies [42]. Interestingly, in clinical trials and case reports, imatinib also ameliorated hemodynamics [11, 12, 16] and restored heart function [13] suggesting that it may also improve vascular tone. Meanwhile, corroborating studies in PAs from healthy or hypertensive rats demonstrated the relaxant effects of imatinib [14, 15]. Clinically, however, effects on PVs may be more relevant since the most common cause of PH is LHD, a condition that primarily affects PVs [8, 43]. Therefore, in the present study we focused on the relaxant effects of imatinib in the pulmonary venous bed. We selected GPs, as the access to human lung tissue is limited and the pulmonary smooth muscle pharmacology of GPs resembles human tissue much better than rats or mice [28, 44]. Most studies were performed using PCLS, a method [28, 29] that allows to study PVs within their natural tissue anatomy [45]. To study the effects of imatinib on the R_post_, we used the double occlusion method [38] in isolated perfused lungs (IPL) of GPs. To mimic a pathophysiological aspect of PH, namely the overexpression of ET-1 and its receptor [46, 47], we pre-constricted PVs with ET-1.

In the present study, imatinib relaxed pre-constricted PVs with an EC_50_ value of 32 μM in line with a previous study in PAs [14]. Imatinib-induced relaxation was dependent on the generation of cAMP and on the activation of K^+^-channels. In the IPL, perfused or nebulized imatinib reduced the ET-1-induced increase of R_post_. Notably, nebulized imatinib might prevent perfusion/ventilation mismatches and imatinib-related systemic side effects such as subdural hematoma or hypotension. Further, PDGF-BB contracts PVs, a finding which might explain in part why imatinib exerts relaxation. Our results suggest the use of imatinib in the therapy of PH due to LHD.

### Mechanisms contributing to imatinib-induced relaxation

The tone of vascular SMCs is regulated by various signaling cascades, which ultimately modulate intracellular Ca^2+^-levels or/and Ca^2+^-sensitivity of the myofilaments. In this context, the nucleotides cAMP and cGMP play a leading part. Cyclic AMP — via activation of PKA — causes relaxation by K^+^-channel-stimulation. Further, cAMP acts in a Ca^2+^-desensitizing manner: 1) cAMP blocks myosin light chain kinase (MLCK); 2) cAMP activates myosin light chain phosphatase (MLCP) [48]. It has been reported that Ca^2+^-desensitization plays a role in imatinib-induced relaxation [14]. Here we show that imatinib leads to increased intracellular cAMP levels in cavine PVs. The functional relevance of this cAMP-increase was shown by inhibiting adenyl cyclase (SQ22536) or PKA (KT5720); both inhibitors attenuated the relaxant effect of imatinib. The generation of cAMP has not yet been reported within the context of imatinib- or TKI-induced vasorelaxation, even though they are supported by own unpublished results from human pulmonary vessels and by Kim et al. [49] who proved in murine interstitial cell of Cajals that the imatinib-induced inhibition of pacemaker potentials depends on the generation of cAMP [49]. These results raise the question, how to explain the imatinib-induced increase of cAMP or more precisely how PDGFR-inhibition is linked to the activation of G_αs,_ the inhibition of G_αi_ or the inhibition of PDE-III: 1) There is evidence that RTKs and GPCR interact together, e.g. via transactivation [50], as it was already shown for EGF and G_αi_ [51]. 2) Recently it was reported that PDGF inhibits regulators of G protein signaling (RGS) [52]. In particular, it was shown that PDGF suppresses the expression of RGS-5 in vascular SMC; since downregulation of RGS-5 activates G_αi_, adenyl cyclase is blocked and the generation of intracellular cAMP reduced.

Cyclic AMP/PKA-signaling appears critical for the imatinib-induced relaxation, although inhibition of adenyl cyclase or PKA did not completely prevent it. This suggests the involvement of other signaling cascades such as NO/cGMP/PKG. PKG promotes Ca^2+^-desensitization via MLCP-activation and stimulates K^+^-channels [48]. The NO/cGMP/PKG-signaling highly depends on an intact endothelium releasing aside NO [53] also prostacyclin [54, 55]. In PCLS, PVs dispose of an intact endothelium [56], even though NO/cGMP/PKG-signaling did not contribute to imatinib-induced relaxation, as 1) imatinib failed to increase cGMP; 2) inhibition of NO-synthesis (L-NAME) or PKG (KT5823) did not attenuate imatinib-induced relaxation. Despite these results, we cannot conclude if imatinib-induced relaxation is endothelium independent or dependent, as it is highly based on cAMP/PKA-signaling and within this context, the endothelial release of prostacyclin might be of impact. Our results corroborate in part those of Abe et al. [14], who showed that imatinib relaxes PAs from hypertensive rats, despite NO-inhibition. Aside that, they [14] proved in endothelial denuded PAs (rats) that the relaxant effect of imatinib does not depend on the endothelium or the endothelial prostacyclin release, as well. In contrast, in SMCs from human corpus cavernosum [57] and in prostatic SMCs [58], imatinib-induced relaxation appears to depend on NO-signaling. Thus, the role of NO/PKG/cGMP in the imatinib-induced relaxation appears to be species and organ-specific.

To study further mechanisms, we evaluated the impact of K_ATP_-, ${\mathrm{BK}}_{\mathrm{Ca}}^{2+}$- and K_v_-channels, all of which are expressed in pulmonary vessels [59, 60]. Activation of K^+^-channels leads to cell membrane hyperpolarization resulting in reduced cytosolic Ca^2+^-influx and in relaxation of vascular SMCs. In GP PVs, inhibition of all three K^+^-channels attenuated imatinib-induced relaxation, with K_ATP_- and K_v_-channel inhibitors being more effective. The minor role of ${\mathrm{BK}}_{\mathrm{Ca}}^{2+}$-channels might be explained by the fact that ${\mathrm{BK}}_{\mathrm{Ca}}^{2+}$-channels dominate in conduit PAs [59], although in rat PAs K^+^-channel-activation could not explain the imatinib-induced relaxation [15]. In line with our results, a dominant role of K_ATP_- versus ${\mathrm{BK}}_{\mathrm{Ca}}^{2+}$-channels was demonstrated for the imatinib-induced relaxation in human prostatic SMCs [58]. Conversely, PDGF has been shown to decrease K_v_-channel currents [61]. Moreover, the imatinib-induced activation of K^+^-channels does not only mediate relaxation, but also contributes to its antiproliferative effects, as hyperpolarization of the cell membrane lowers the intracellular Ca^2+^-influx/content and counteracts the vascular remodeling [59]. A similar relationship has also been shown for the Ca^2+^-sensitizer levosimendan that develops pulmonary vasorelaxant effects [29] and antiproliferative properties via K^+^-channel-activation [62]. Finally, imatinib-induced relaxation is based on cAMP/PKA dependent mechanisms and on K^+^-channel activation. Both signaling pathways may interact to some extent in an additive manner [48, 63].

### Pulmonary vasorelaxant effects of perfused and nebulized Imatinib in the IPL

The resistance of the pulmonary venous bed, precisely the postcapillary resistance (R_post_) is increased in PH due to LHD and mainly responsible for the generation of hydrostatic edema [6]. Vice versa, reduction of R_post_ is one of the major therapeutic goals in this disease. To address the vasorelaxant effects of imatinib on R_post_ *in situ*, we perfused imatinib in isolated lungs. This set-up enables: 1) to address the pulmonary arterial and venous segment independent from each other; 2) to monitor P_PA_, P_LA_ and the capillary pressure (P_cap_) and 3) to calculate R_post_ from P_LA_ and P_cap_. In order to access the resistance of the pulmonary venous bed, the calculation of R_post_ is highly relevant, because it includes the small PVs. Further, it is superior to the P_pcw_, which rather reflects the pressure in large PVs [64] or P_LA_ [65].

Perfusion of 20 nM ET-1 significantly increased P_PA_, R_pre_ and R_post_ in the IPL. In contrast, P_LA_ did not change, as it is expected due to the reservoir function of the left atrium in this model. In the present study, perfusion of 10 μM imatinib significantly lowered R_post_. This finding is in line with the results of the IMPRES study that showed improved hemodynamics after the application of imatinib, expressed as a decrease in PVR and an increase in the 6 min walk distance [16]; of note, the IMPRES study only included patients with PAH [16]. Our results are further supported by Shah et al. [13], who found that imatinib improves right but also left heart function. However, the IMPRES study [16], as well as the long-term extension of the IMPRES study [17] further revealed undesirable imatinib-related side effects such as vomiting, nausea or peripheral edema that decrease patient compliance; and even more critical were the occurrence of subdural hematoma, QTc prolongation, cardiac failure and syncope, the latter two most probably being caused by systemic hypotension [16, 17]. Unfortunately, however in PH, systemic hypotension is quite problematic, as it decreases the coronary perfusion pressure and consequently worsens left and right ventricular function [66]. To find a way to prevent systemic side effects, we evaluated the possibility and efficacy to apply imatinib locally via the airways [18], as it is already usual in PH for NO-donors, prostanoids or PDE-inhibitors [67, 68]. In the IPL, we could show that nebulized imatinib reduces the ET-1-induced increase of R_post_. The results from the IPL (perfusion/nebulization) correspond to our data from PCLS, even though nebulization of imatinib was less effective in reducing R_post_ than perfusion. As mentioned above (methods), these differences are probably explained by the maximal imatinib up-take: the imatinib up-take via nebulization is 0.71 mg corresponding to 2 mg/kg body weight imatinib and via perfusion 1.18 mg corresponding to 3.5 mg/kg body weight imatinib (the ratio is 0.6). Other effects may also play a role such as an insufficient imatinib-uptake through the alveo-capillary membrane or the degradation of imatinib by intraalveolar macrophages. Here, we performed an IPL model; thus the extent to which nebulized imatinib provokes systemic adverse effects remains to be shown. In summary, we focused on central PVs in PCLS and we addressed R_post_ (IPL)_,_ resembling the peripheral part of the pulmonary venous bed [69, 70]. In both models, we demonstrated that imatinib relaxes the pulmonary venous bed of GPs.

### The role of PDGFR-β and interaction of ET-1 with PDGFR

The antiproliferative effects of imatinib are attributed to inhibition of the tyrosine kinase activity of PDGFR [20]. Beyond that, PDGFR activation is known to enhance the tone of various vessel types from different species by the increase of intracellular Ca^2+^ [26, 71]. So far it is unknown, if imatinib-induced relaxation is linked to PDGFR-inhibition, and further if PDGF also contracts pulmonary vessels. Here, we showed that PDGF-BB contracts PVs and that this contraction is completely prevented by the unselective PDGFR-αβ-inhibitor imatinib. PDGFR-β-inhibition (SU6668) abolished PDGF-BB-induced contraction, whereas PDGFR-α-inhibition (ponatinib) reduced it only mildly and non-significantly. In general, PDGF-BB binds to PDGFR-αα, αβ or ββ [23]. Hence, our results suggest 1) a leading role of PDGFR-ββ or/and PDGFR-αβ, as inhibition of the β-subunit completely prevented PDGF-BB-induced contraction and 2) a minor role of PDGFR-αα, as inhibition of the α-subunit did not prevent contraction. SU6668 has multiple targets such as Aurora kinases, TBK1 and the RTK VEGFR-2, FGFR-1, EGFR or FLK-1/KDR [35]. SU6668 (5 μM) blocks VEGFR-2 (IC_50_ 0.34–2.43 μM) and FGFR-1 (IC_50_ 1.2–10 μM) only moderately, whereas ponatinib (100 nM) potently blocks VEGFR-2 (IC_50_ 1.5 nM) and FGFR-1 (IC_50_ 2.2 nM). Hence, if the inhibition of VEGFR-2 or FGFR-2 would prevent the PDGF-BB-induced contraction, ponatinib should be more effective than SU6668. In addition, the complete inhibition of EGFR (IC_50_ >100 nM) or FLK-1/KDR (IC_50_ 2.1 μM) would require SU6668 concentrations above 5 μM. These considerations support the idea that the contractile effect of PDGF-BB is prevented by inhibition of PDGFR-β [32–37, 72–75]. Conversely, the PDGFR-β inhibitors SU6668 and DMPQ relaxed pre-constricted PVs, whereas the PDGFR-α inhibitor ponatinib had no effect. Further, after inhibition of PDGFR-β by SU6668 or DMPQ (both 5 μM), imatinib only relaxed pre-constricted PVs at 100 μM. These data suggests that imatinib-induced relaxation depends on PDGFR-β inhibition. Thus, our findings suggest that PDGFR-β antagonism does not only attenuate the establishment of PH [10, 76], but also mediates vasorelaxation. Our results are supported by Shiba et al. [77] who proved in cerebral arteries that imatinib counteracts PDGFR-signaling and prevents vasospasm. In an effort to identify a link between ET-1- and PDGFR signaling, we studied if activated ET-1 receptors interact anyhow with PDGFR. To this end, we inhibited PDGFR prior to the treatment with ET-1. Neither inhibition of PDGFR (imatinib), PDGFR-β (SU6668) nor PDGFR-α (ponatinib) did reduce ET-1-induced contraction. Hence, it appears that stimulation of ET-1 receptors does not activate PDGFR or it takes place, but without being sufficient to mediate contraction, possibly because ligand-binding is missing. Recently, Harada and coworkers [78] could show in rat L6 myoblasts that stimulation of ET-1 receptors activates PDGFR downstream signaling by an unknown mechanism. However, they did not study ET-1-induced phosphorylation of PDGFR [78].

Irrespective from a possible interaction of ET-1 and PDGFR, the relaxant effect of imatinib is not limited to ET-1, as it relaxed PAs pre-constricted with U46619 [14, 15], serotonin [14] or L-NAME [15]. Further, imatinib exerts relaxant effects in different tissues, i.e. systemic vessels [15], prostatic tissue [58], PAs [14, 15], corpus cavernosum [57], myometrium [79] and stomach [80] from humans, rabbits, GPs, rats or sheep. Thus, imatinib-induced relaxation appears to be a widespread phenomenon. Beyond that, other TKIs also exert pulmonary vascular relaxant effects (nilotinib/sorafenib) [14] or prevent experimental pulmonary vascular remodeling by PDGFR-inhibition (nilotinib/dasatinib). Within this context, dasatinib plays a particular role, as it also inhibits Src kinases playing a pivotal role in pulmonary arterial remodeling [81, 82]. That is probably why; dasatinib reverses experimental PH even more potently than imatinib [81]. However in patients with CML, dasatinib also provoke drug-induced PAH by an unknown mechanism [83, 84]. Fortunately, this dasatinib-associated adverse effect appears to be reversible, rare and limited to dasatinib [85–88].

## Conclusions

Imatinib relaxes PVs by activation of cAMP/PKA and potassium channels (K_ATP_-, ${\mathrm{BK}}_{\mathrm{Ca}}^{2+}$-, K_v_-channels), mechanisms that are related in an unknown way to the inhibition of PDGFR-β. The clinical importance of imatinib-induced relaxation is supported by reports that imatinib improved the clinical condition of a patient with pulmonary venous occlusive disease within 24 h [12] and ameliorated PH due to congenital hernia [11]. The prompt improvement in both cases favors relaxant effects of imatinib in contrast to the reversal of vascular remodeling which lasts longer [10]. However, until now studies with human pulmonary vessels are still missing and quite difficult to obtain. Our data suggest a beneficial role of imatinib in PH due to LHD. Since imatinib combines short-term relaxant, even if inhaled and long-term antiproliferative effects, it may represent a promising approach to treat PH due to LHD.

## Abbreviations

GPs: Guinea pigs
IPL: Isolated perfused lungs
LHD: Left heart disease
LMM: Linear mixed model analysis
MLCK: Myosin light chain kinase
MLCP: Myosin light chain phosphatase
PAH: Pulmonary arterial hypertension
PAs: Pulmonary arteries
P_cap_: Capillary pressure
PCLS: Precision-cut lung slices
PDGF: Platelet derived growth factor
PDGFR: PDGF-receptor
PH: Pulmonary hypertension
P_LA_: Left atrial pressure
P_PA_: Pulmonary arterial pressure
P_pcw_: Pulmonary capillary wedge pressure
PVH: Pulmonary venous hypertension
PVR: Pulmonary vascular resistance
PVs: Pulmonary veins
RGS: Regulators of G protein signaling
R_post_: Postcapillary resistance
R_pre_: Precapillary resistance
RTK: Receptor tyrosine kinase
SMCs: Smooth muscle cells
TKI: Tyrosine kinase inhibitor
TPG: Transpulmonary gradient (TPG)
