# Erratum to: Imatinib relaxes the pulmonary venous bed of guinea pigs

**DOI:** 10.1186/s12931-017-0612-z

**Published:** 2017-06-19

**Authors:** Nina A. Maihöfer, Said Suleiman, Daniela Dreymüller, Paul W. Manley, Rolf Rossaint, Stefan Uhlig, Christian Martin, Annette D. Rieg

**Affiliations:** 1 Institute of Pharmacology and Toxicology, Medical Faculty Aachen, RWTH-Aachen, Aachen, Germany; 2 Novartis Pharma, AG Basel, Switzerland; 3 Department of Anesthesiology, Medical Faculty Aachen, RWTH-Aachen, Aachen, Germany

## Erratum

Upon publication of the original article [1], it was noticed that the section Authors’ contributions was incorrectly given. The section Authors’ contributions should read as, “NAM performed the experiments, analysed the data, interpreted the data and wrote the manuscript. SS performed the experiments, analysed the data and interpreted the data. DD performed the experiments, interpreted the data and critically reviewed the manuscript. PWM analysed the data, interpreted the data and critically reviewed the manuscript. RR analysed the data, interpreted the data and critically reviewed the manuscript. SU analysed the data, interpreted the data and critically reviewed the manuscript. CM designed the study, analysed the data, interpreted the data and critically reviewed the manuscript. ADR designed the study, performed the experiments, analysed the data, interpreted the data and CRITICALLY REVIEWED AND REVISED THE MANUSCRIPT. All authors read and approved the final manuscript. ” This has now been acknowledged and corrected in this erratum.
